# Haplotype analysis incorporating ancestral origins identified novel genetic loci associated with chicken body weight using an advanced intercross line

**DOI:** 10.1186/s12711-024-00946-y

**Published:** 2024-12-20

**Authors:** Lina Bu, Yuzhe Wang, Lizhi Tan, Zilong Wen, Xiaoxiang Hu, Zhiwu Zhang, Yiqiang Zhao

**Affiliations:** 1 State Key Laboratory of Animal Biotech Breeding, College of Biological Sciences, China Agricultural University, Beijing, China; 2 National Research Facility for Phenotypic and Genotypic Analysis of Model Animals (Beijing), China Agricultural University, Beijing, China; 3 Department of Crop and Soil Sciences, Washington State University, Pullman, WA USA

## Abstract

**Background:**

The genome-wide association study (GWAS) is a powerful method for mapping quantitative trait loci (QTL). However, standard GWAS can detect only QTL that segregate in the mapping population. Crossing populations with different characteristics increases genetic variability but F2 or back-crosses lack mapping resolution due to the limited number of recombination events. This drawback can be overcome with advanced intercross line (AIL) populations, which increase the number recombination events and provide a more accurate mapping resolution. Recent studies in humans have revealed ancestry-dependent genetic architecture and shown the effectiveness of admixture mapping in admixed populations.

**Results:**

Through the incorporation of line-of-origin effects and GWAS on an F_9_ AIL population, we identified genes that affect body weight at eight weeks of age (BW8) in chickens. The proposed ancestral-haplotype-based GWAS (testing only the origin regardless of the alleles) revealed three new QTLs on GGA12, GGA15, and GGA20. By using the concepts of ancestral homozygotes (individuals that carry two haplotypes of the same origin) and ancestral heterozygotes (carrying one haplotype of each origin), we identified 632 loci that exhibited high-parent (the heterozygote is better than both parents) and mid-parent (the heterozygote is better than the median of the parents) dominance across 12 chromosomes. Out of the 199 genes associated with BW8, *EYA1*, *PDE1C*, and *MYC* were identified as the best candidate genes for further validation.

**Conclusions:**

In addition to the candidate genes reported in this study, our research demonstrates the effectiveness of incorporating ancestral information in population genetic analyses, which can be broadly applicable for genetic mapping in populations generated by ancestors with distinct phenotypes and genetic backgrounds. Our methods can benefit both geneticists and biologists interested in the genetic determinism of complex traits.

**Supplementary Information:**

The online version contains supplementary material available at 10.1186/s12711-024-00946-y.

## Background

Growth is a highly polygenic trait and one of the most important economic traits for chickens (*Gallus gallus*) [1]. The chicken quantitative trait loci (QTL) database lists over 2200 growth-related QTL across the genome, mainly on *Gallus gallus* (GGA) chromosomes 1, 2, 3, 4, and Z [2]. While genetic mapping and QTL analysis can be performed in any population, it is harder to detect associations signals in populations under selection because of allele fixation [3]. In contrast, an advanced intercross line (AIL) population, created by sequential random intercrossing of ancestors with distinct phenotypes and genetic backgrounds, offer better association detection thanks to large genetic variability and increased recombination density compared to F2 crosses [4]. In our recent studies, we identified significant QTL on GGA1 and GGA27 for body weight based on an AIL population built from two divergent populations for weight: a High-Quality Chicken Line A (HQLA) and a Huiyang Bearded Chicken (HB) population [5, 6].

The traditional strategy of genetic mapping for growth-related traits relies on single nucleotide polymorphism (SNP)-based genome-wide association studies (GWAS). With advances in sequencing technology and statistical modeling, accurate haplotype information now is easier to obtain [7]. Haplotype-based GWAS is biologically more meaningful than SNP-based GWAS because it has the advantage of combining linked SNPs to control false positives and capture short-range interactions [8–11]. However, the heterogeneous haplotype structure in a population often results in reduced statistical power because of excessive degrees of freedom in haplotype-based analyses [12]. To address this issue, parsimonious approaches have been employed to group haplotypes into few clusters based on sequence similarity, enhancing statistical power [13, 14].

Population stratification due to genetic ancestry can result in spurious associations in GWAS. To counter this, strategies such as fitting principal components as covariates in statistical models have been used [15, 16]. However, the AIL population, which is genetically highly mixed due to recombination (ancestral admixture) over many generations, is less affected by population stratification than natural populations. Considering the contribution of ancestry-specific variations to phenotype, many studies have utilized ancestry information to infer ancestry-phenotype correlations [17–21], to leverage local ancestries for the detection of epistasis [22, 23], and to improve breeds [23]. In our previous GWAS using an F_9_ AIL population, analysis suggested distinct effects of haplotypes of different ancestral origins. These efforts revealed ancestry-dependent genetic architecture and contributions in admixed populations.

The primary objective of this study was to extend our previous studies to identify genes significantly associated with body weight. To adequately employ the characteristics of the AIL population, we proposed an ancestral-haplotype-based GWAS, incorporating ancestral information into haplotype association tests. Additionally, we applied the concepts of ancestral homozygotes and ancestral heterozygotes to analyze ancestry-based dominance, encompassing high-parent and mid-parent dominance. Our results shed light on a better utilization of the AIL population for genetic mapping.

## Methods

### The AIL population

The AIL population analyzed in this study was generated by crossing a High-Quality Chicken Line A (HQLA) with a Huiyang Bearded Chicken (HB). The HQLA population was created by crossing the commercial Anak Broiler breed with a Chinese chicken line, while the HB population is an indigenous Chinese breed. At eight weeks of age, the body weight of HQLA is three times greater than that of HB. The AIL generations (F_3_ to F_9_) were produced by random mating following the F_2_ generation. For a detailed description of the construction and phenotypic data of the AIL population, please refer to previous publications [5, 24]. The phenotype analyzed in this study was body weight at eight weeks of age (BW8) in the F_9_ generation.

### Genotyping and haplotyping

Genotyping-by-sequencing (GBS) data of the F_0_, F_8_, and F_9_ generations were used in this study. For the F_9_ generation, double-enzyme (*Eco*RI/*Mse*I) GBS libraries were prepared, and sequencing was performed on an Illumina Nextseq500 sequencer. On average, each sample sequenced by the GBS method produced 3.44 million high-quality barcoded reads [25]. Genome-wide SNPs were identified using the TASSEL GBS analysis pipeline (version 5.2.31) [26] with GRCg6a (released 2018) as the reference genome. SNP quality filtering was carried out using VCFtools (version 0.1.16) [27] with the criteria of minor allele frequency > 0.01, genotypes with quality > 98, sequencing depth > 4, max missing rate < 0.2, and biallelic loci only. Finally, 189,401 GBS SNPs (GGA1-GGA28) for 16 HQLA, 14 HB, 185 F_8_, and 585 F_9_ individuals were retained.

To perform haplotype phasing and imputation, we tried Beagle 5.0 [28] and SHAPEIT 2.0 [29] software, both without external reference panel. Since SHAPEIT accounts for familial relationships, we first used the GTOOL software (https://www.well.ox.ac.uk/~cfreeman/software/gwas/gtool.html) to convert ped/map files into gen/sample files (gtool -P --ped file.ped --map file.map --og file.gen --os file.sample) to add parent–child information, based on pedigree information of the F_8_ (parental generation) and F_9_ generations. Haplotype phasing and imputation were performed using Beagle 5.0 and SHAPEIT 2.0 with the following parameters: beagle.jar gt=file.vcf out=file phased gp =true impute=true; shapeit -G file.gen file.sample -O file.phased --force —duohmm. Consistency of the two software programs was assessed based as the proportion of identically phased genotypes and was found to be around 90% (see Additional file 1: Table S1). In the end, we used Beagle since it is simpler and faster.

Based on our previous study [5], linkage disequilibrium based on r^2^ decayed rapidly in the F_9_ population, and average physical distance when r^2^ equals 0.1 was 27 Kb. There were approximately five SNPs for 27 Kb physical distance in our genotype data. Thus, the genome was subsequently divided into blocks of five successive SNPs and haplotype alleles for each block were retrieved.

### Construction of genetic map

The LEP-MAP3 software [30] was used to construct the genetic map based on pedigree information of the F_8_ (parental generation) and F_9_ generations. For each chromosome, all markers were sorted by physical location. Parental genotypes were first called using the ParentCall2 module of the LEP-MAP3 software. The Filtering2 module was used to remove non-informative and distorted markers, with parameters set to removeNonInformative = 1 and dataTolerance = 0.0000001. The SeparateChromosomes2 module was then used to categorize markers into linkage groups (LG), with parameters set to lodLimit = 5. Lastly, markers clustered into corresponding linkage groups were ordered using the OrderMarkers2 module.

### Haplotype diversity and dissimilarity statistics

The H12 statistic [31] is a commonly used haplotype diversity measure that is based on the sum of the squares of haplotype frequencies, combining the two most common haplotypes into a single frequency. We also extended the concept of H12 to H123 and H1234, which combine the three or four most common haplotypes into a single frequency. H12, H123, and H1234 were computed as: $H12={({p}_{1}+{p}_{2})}^{2}+{\sum }_{i=3}^{\infty }{{p}_{i}}^{2}, H123={({p}_{1}+{p}_{2}+{p}_{3})}^{2}+{\sum }_{i=4}^\infty {{p}_{i}}^{2}$, $H1234={({p}_{1}+{p}_{2}+{p}_{3}+{p}_{4})}^{2}+{\sum }_{i=5}^\infty {{p}_{i}}^{2}$, where *p*_*i*_ is the frequency of haplotype *i*, with ${\sum }_{i=1}^\infty {p}_{i}$=1 and *p*_*1*_ ≥ *p*_*2*_ ≥ $\cdots$ ≥ *p*_*i*_ [31].

Jaccard distance [32] measures dissimilarity between two populations (A, B), it is computed by using the formula $\text{JD}=1-\frac{|\text{A}\cap \text{B}|}{|\text{A}\cup \text{B}|}$; where A and B represent haplotype alleles in two populations. Jensen-Shannon divergence [33] measures dissimilarity between two probability distributions (A, B) and is calculated as: $\text{JSD}=\frac{1}{2}\text{KL}(\text{A}||\text{M})+\frac{1}{2}\text{KL}(\text{B}|\left|\text{M}\right)$; where $\text{M}=\frac{1}{2}(\text{A}+\text{B})$, $\text{KL}(\text{A}||\text{M})=\sum_{\text{x}}\text{A}\left(\text{x}\right)\text{log}\frac{\text{A}\left(\text{x}\right)}{\text{M}\left(\text{x}\right)}$ and $\text{KL}(\text{B}||\text{M})=\sum_{\text{x}}\text{B}\left(\text{x}\right)\text{log}\frac{\text{B}\left(\text{x}\right)}{\text{M}\left(\text{x}\right)}$, with the probability distributions being the haplotype allele frequency distributions of the two populations. Bray–Curtis dissimilarity [34] measures dissimilarity between two populations based on counts, using $\text{BCD}=1-2\frac{\sum \text{min}({\text{S}}_{\text{A},\text{i}},{\text{S}}_{\text{B},\text{i}})}{\sum {\text{S}}_{\text{A},\text{i}}+\sum {\text{S}}_{\text{B},\text{i}}}$, where ${\text{S}}_{\text{A},\text{i}}$ and ${\text{S}}_{\text{B},\text{i}}$ are the counts of the haplotype alleles in populations A and B, respectively. Custom Perl scripts were used to calculate H12, H123, H1234 and Jaccard distance and the SciPy python package (https://scipy.org) was used to compute the Jensen-Shannon divergence and Bray–Curtis dissimilarity between HQLA or HB and the F9 population.

### Ancestral inference and coding

The RFMix software [35] was used to infer the local ancestry for each haplotype of each F_9_ individual, utilizing the genetic map constructed above. RFMix partitions each chromosome into windows and infers local ancestry within each window by employing a conditional random field (CRF) approach parameterized by random forests trained on reference panels. The CRF is an undirected probabilistic graphical model that is commonly applied to sequence labeling and segmenting problems. RFMix further uses maximum-a-posteriori (MAP) estimation or smoothing to refine ancestry assignments. Here, the HB and the HQLA populations were used as the reference panels to infer the local ancestry for F_9_ individuals. The CRF spacing was set to five SNPs. According to the outputs of RFMix, we took the probability value of 0.5 as the threshold to assign the origin from which a haplotype was derived. I.e., if the probability of haplotypes originating from the HQLA population was greater than or equal to 0.5, the haplotype was considered to be of HQLA origin and coded as 1; otherwise, the haplotypes were considered to be of HB origin and coded as 0.

### SNP and haplotype-based genome-wide association analyses

For the SNP-based GWAS for the F_9_ generation, we applied the standard mixed linear model analysis (MLMA) method implemented in the GCTA software [36]. The model can be written as: $\mathbf{y}=\mathbf{Q}{\varvec{\upalpha}}+\mathbf{x}\upbeta +\mathbf{g}+\mathbf{e}$, where $\mathbf{y}$ is the vector of BW8 phenotypes of the F_9_ individuals, $\mathbf{Q}$ is the design matrix for covariates, including sex and batch; ${\varvec{\upalpha}}$ is the vector of effects for the covariates; $\mathbf{x}$ is the vector of genotype indicator variables, coded as 0, 1, or 2; $\upbeta$ is the SNP effect to be tested for association; $\mathbf{g}$ is the vector of polygenic effects captured by the genetic relationship matrix (GRM), which was calculated using all SNPs; and $\mathbf{e}$ is the vector of residuals. Associations with a false discovery rate (FDR) [37] ≤ 0.05 were considered significant.

The above mixed model was modified for the ancestral-haplotype-based GWAS by coding diploid individuals in the F_9_ population with haplotypes of ancestral origins HB/HB, HB/HQLA, HQLA/HQLA as 0, 1, and 2, respectively, representing the number of copies of HQLA ancestry (see above). The GRM was thus calculated using all ancestry-coded haplotypes, accounting for global ancestry. The ancestral-haplotype-based GWAS was then performed using GCTA in the same way as the SNP-based GWAS.

We conducted the haplotype-based GWAS separately using the lme4qtl R package [38]. For each haplotype block, we coded the haplotype combination as categorical variables for each individual and tested one block at a time. The model can be written as: $\mathbf{y}=\mathbf{Q}{\varvec{\upalpha}}+{\mathbf{X}}_{{\varvec{h}}}{\varvec{\upbeta}}+{\mathbf{g}}_{{\varvec{h}}}+\mathbf{e}$, where ${\mathbf{X}}_{{\varvec{h}}}$ is the design matrix for haplotype combinations as factors; ${\varvec{\upbeta}}$ is the vector of effect size of haplotype combinations; ${\mathbf{g}}_{{\varvec{h}}}$ is the vector of polygenic effects captured by the GRM calculated using all haplotypes, computed as described below, and all other variables are the same as for the SNP-based GWAS model. To assess the overall statistical significance of each haplotype block, we fitted a null model that is the same as the alternative model, except it does not include ${\mathbf{X}}_{{\varvec{h}}}{\varvec{\upbeta}}$. After that, we used the lme4qtl::update function to add the haplotypes as fixed effect into the null model for each block. ANOVA was then applied to test the difference between the alternative model and the null model.

The haplotype-based GRM was calculated referring to method 1 described in [39]. In short, the genome was divided into *n* segments using a five-SNPs window. Given a population of N individuals, each block contains 2N haplotype alleles for diploid individuals. We assigned a score of 1 when two haplotype alleles were the same and 0 when they differed. This process generated a 2N*2N matrix, **Γ**_***i***_, for each block *i*. The final haplotype relationship matrix ${\varvec{\Gamma}}$ was obtained by summing up the segmental matrices and dividing by n, as follows: ${\varvec{\Gamma}}=\sum_{i=1}^{n}{{\varvec{\Gamma}}}_{{\varvec{i}}}/n$. The 2N*2N matrix was converted to the N*N haplotype-based GRM at the individual level using $\mathbf{H}=\mathbf{K}{\varvec{\Gamma}}\mathbf{K}^{\prime}/2$, where $\mathbf{K}=\mathbf{I}\otimes [\mathbf{11}]$ ($\mathbf{I}$ is an *m* by *m* identity matrix, where *m* is the number of individuals, and ⊗ is the Kronecker product). In haplotype-based GWAS, the haplotype-based GRM was constructed once, while the parameters were estimated every time the model was executed for each haplotype block.

### Block-wise haplotype analysis for effect size estimation

Both haplotype-based GWAS and ancestral-haplotype-based GWAS assessed the overall statistical significance of each haplotype block. For a given significant haplotype block, the effect size of haplotype alleles was estimated with a different mixed model, using the hglm R package [40]. This model can be written as: $\mathbf{y}=\mathbf{Q}{\varvec{\upalpha}}+{\mathbf{Z}}_{{\varvec{d}}}{\varvec{\upmu}}+\mathbf{e}$**,** where ${\mathbf{Z}}_{{\varvec{d}}}$ is the dosage matrix containing counts of different haplotype alleles for each individual, with the sum of elements in each row equaling 2 (representing diploid status); ${\varvec{\upmu}}$ is the vector of random effects for each haplotype allele, and all other variables are as defined previously. To estimate the effect size of haplotype alleles in haplotype-based GWAS, haplotype alleles in each block were directly incorporated in the model. For ancestral-haplotype-based GWAS, effect was estimated for each haplotype allele of each origin. Dosage coding was as described in the following.

Consider a population comprising two individuals, where a haplotype block consists of two haplotype alleles (hap1, hap2). We assume the first individual is homozygous for hap1, the second individual is heterozygous, i.e., has one hap1 and one hap2. Then, the haplotype matrix is coded as follows:

$${\mathbf{Z}}_{{\varvec{d}}}= \left[\begin{array}{lll}2 & 0 \\ 1 & 1 \end{array} \right].$$

By incorporating ancestral origins, each haplotype allele can have two ancestral labels: hapl_HB, hap1_HQLA, hap2_HB, and hap2_HQLA. Let us assume that the two hap1 alleles of the first individual originated from different ancestral origins, while for the second individual, hap1 is from the HQLA population and hap2 is from the HB population. When coding haplotype alleles with ancestral labels, the haplotype matrix ${\mathbf{Z}}_{{\varvec{d}}}$ will then become:

$${\mathbf{Z}}_{{\varvec{d}}}=\left[\begin{array}{cc}1& \begin{array}{cc}1& \begin{array}{cc}0& 0\end{array}\end{array}\\ 0& \begin{array}{ccc}1& 1& 0\end{array}\end{array}\right].$$

### Ancestry-based dominance analysis

In addition to additive effects, dominance (interaction between alleles at the same locus) can also be involved in the determination of the phenotype. To detect their influence, we firstly corrected the phenotypes of F_9_ individuals to account for the effects of sex and batch, and the residuals from that model were used as new phenotypes for further analyses.

For each individual in the F_9_ population and for each block, we defined the ancestral heterozygotes (HB/HQLA) as consisting of one haplotype from HB and one from from HQLA, while ancestral homozygotes were defined as having both haplotypes from HB (HB/HB) or both from HQLA (HQLA/HQLA). Ancestry-based dominance was estimated in two different settings: high-parent dominance was defined as the ancestral heterozygote exhibiting significantly higher body weight than both ancestral homozygotes, while mid-parent dominance was defined as the ancestral heterozygote exhibiting significantly higher body weight than the median of the ancestral homozygotes. A non-parametric Kruskal–Wallis test was initially conducted for each block to assess the hypothesis that the medians of BW8 between ancestral homozygotes and ancestral heterozygotes are equal. When this null hypothesis was rejected, Steel–Dwass post-hoc tests were employed to determine which one significantly differed from others within the block. A false discovery rate [37] FDR ≤ 0.05 was considered as significant.

### Gene annotation and candidate gene prioritization

To obtain candidate genes for body weight, genes that overlapped with significant GWAS loci were retrieved according to coordinates recorded in the chicken genomic general transfer format (GTF) files from Ensembl. To prioritize candidate genes reported by GWAS and by dominance analyses, we assembled a set of 322 genes associated with human body weight from the GWAS Catalog (https://www.ebi.ac.uk/gwas/) as training genes (see Additional file 2 Table S2). Then, the ToppGene [41] web service (https://toppgene.cchmc.org/) was used to perform gene prioritization based on functional similarity to the training genes.

## Results

### Haplotype structures of the HB and HQLA populations

**Fig. 1:**
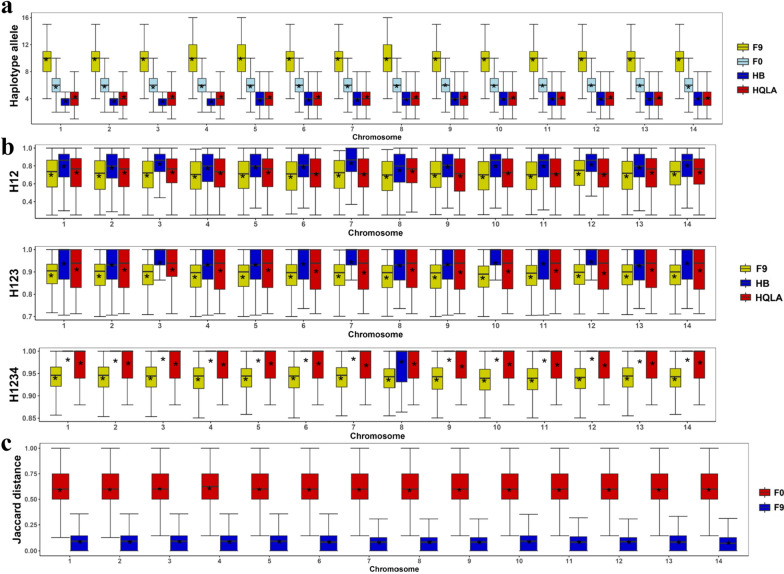
Comparison of haplotype structure between the F_0_ and F_9_ populations (GGA1-GGA14). **a** Counting unique haplotypes in different populations. **b** Distribution of H12, H123, and H1234 statistics in different populations. **c** Distribution of Jaccard distance of F_0_ and F_9_ populations "*" indicates the mean value

For phased data, the genome was divided into non-overlapping blocks of five successive SNPs. Haplotype alleles within each block were counted for each population across the chromosomes. As shown in Fig. 1a and Additional file 3: Fig. S1a, the number of haplotype alleles per block was similar between HB and HQLA populations, with an average of four. In contrast, the average number of that in the F_0_ generation (HB + HQLA) was six for most chromosomes, indicating that 33% of haplotype alleles were shared between the two populations. The average number of alleles per haplotype in the F_9_ population was 10 for each chromosome and block. This suggests that the population has generated 67% more haplotypes due to genetic recombination or/and mutations since hybridization.

To quantify haplotype diversity, H12 statistics were calculated for each population. The results were consistent with the counts of unique haplotypes, with the F_9_ population exhibited the lowest H12 value, indicating the highest haplotype diversity. Compared to the counts of haplotype alleles, the quantitative H12 approach offered higher resolution and differentiated HB from HQLA, having relatively lower diversity (see Fig. 1b and Additional file 3: Fig. S1b). We extended the H12 statistics to H123 and H1234 by considering more haplotypes with a higher frequency, which indicated that the F_9_ population showed the highest haplotype diversity. The H1234 values for the F_9_, HB, and HQLA populations were 0.914, 0.969 and 0.944, respectively.

On the basis of the haplotype structure of the HB and HQLA populations, we further measured the haplotype difference between populations using Jaccard distance. The Jaccard distance between HB and HQLA population was much higher compared to their differences from the F_9_ population, as well as from random sets drawn from the F_9_ population (see Fig. 1c, Additional file 3: Fig. S1c). To better quantify these distances, we recalculated the haplotype difference using Jensen-Shannon divergence and Bray–Curtis distance, which consider haplotype frequencies in addition to haplotype alleles. As shown in Table 1, consistent results were obtained and clearly showed the differentiation and heterogeneity of haplotypes in the HB and HQLA populations. The F_9_ population was, however, genetically well-mixed and homogeneous, consistent with our previous study [5].

**Table 1:** Differences in haplotype frequencies between populations

Method/′populations	HQLA_HB	HQLA_F_9_	HB_F_9_	F_9__F_9_
Jensen-Shannon divergence	0.227	0.087	0.093	0.004
Bray–Curtis distance	0.459	0.265	0.266	0.041

HQLA_HB, HQLA_F_9_, HB_F_9_, and F_9__F_9_ represent the differences in haplotype frequencies between the pair of populations, respectively

Given that the haplotypes in F_9_ were originally inherited from the HB and HQLA populations and haplotype diversity increased through recombination, we constructed a genetic map of the hybrid population to facilitate further analysis, using pedigree and genetic information from the F_8_ and F_9_ generations (see Additional file 4: Table S3). Our genetic map for the 28 autosomes spanned about 2644 cM (see Additional file 5: Table S4), which was shorter than 3016 cM previously reported for chicken [24]. Consistent with previous findings, the recombination rate of small chromosomes was significantly greater than that of large chromosomes, and the map of females was longer than that of males (see Additional file 5: Table S4).

### Novel associations identified by ancestral-haplotype-based GWAS

**Fig. 2:**
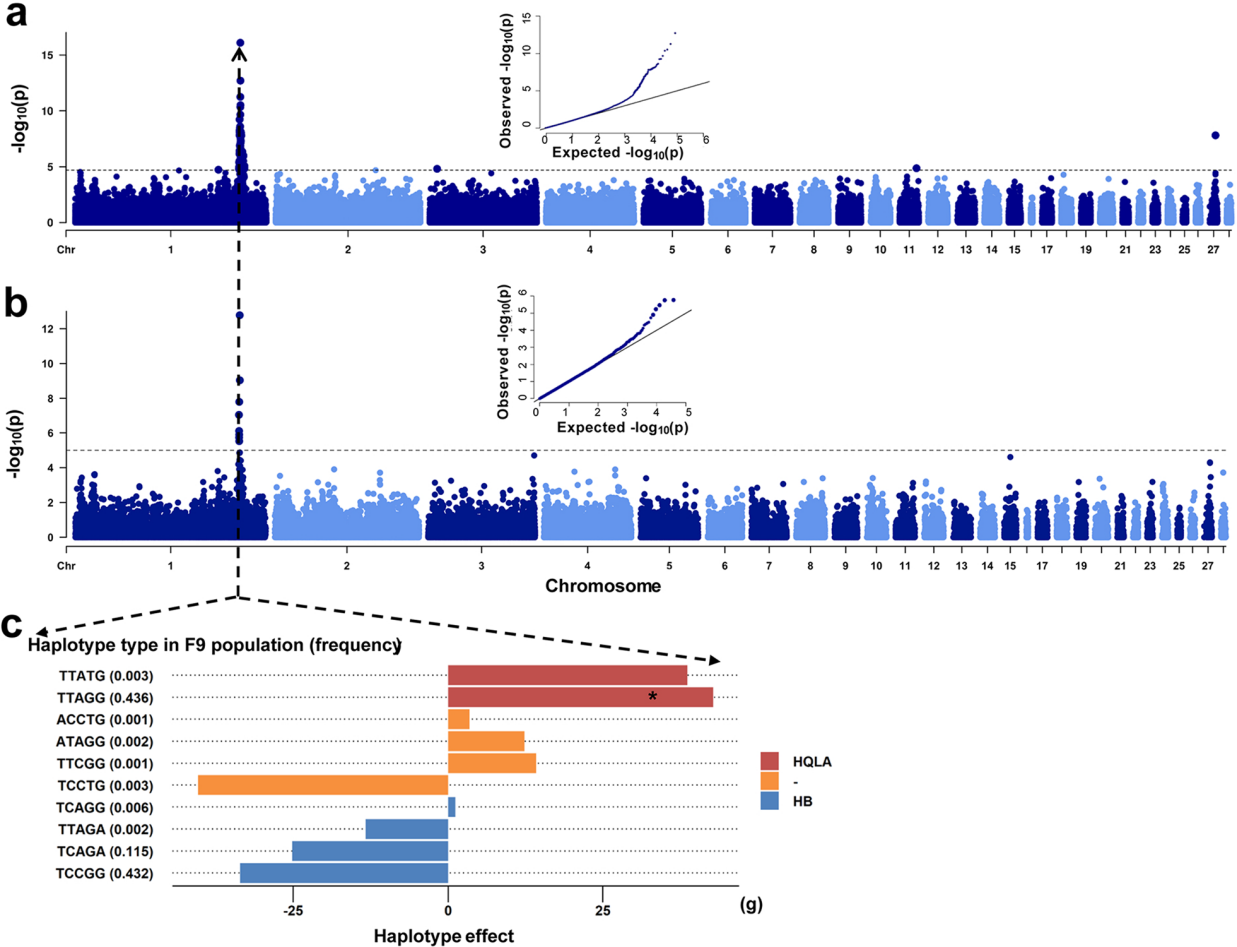
Genome-wide association analysis based on SNPs and haplotypes. **a** Results from SNP-based GWAS (The dashed black horizontal line shows the FDR < 5% cutoff). **b** Results from haplotype-based GWAS (The dashed black horizontal line shows the FDR < 5% cutoff). **c** Estimates of effect sizes of haplotypes for the most significant locus (GGA1: 170,559,701) in the F_9_ population. The red bar indicates haplotypes exclusive to the HQLA population, the blue bar indicates haplotypes exclusive to the HB population, and the orange bar indicates haplotypes absent in both populations. “*” indicates that the TTAGG haplotype is present in both HB and HQLA populations, but the haplotype frequency is much higher in the HQLA population (0.969) compared to the HB population (0.036)

Standard SNP-based GWAS for BW8 was first performed for the F_9_ population using a mixed model implemented in the GCTA software (see Additional file 6: Table S5). A significant QTL region (169.6–173.6 Mb) was identified on GGA1 (Fig. 2a and Additional file 7: Table S6), consistent with the previous report [6]. As haplotypes are more genetically informative, we used five successive SNPs to form haplotype blocks. Each individual was coded by its haplotype combination and haplotype-based GWAS was performed (see Methods and Additional file 8: Table S7). The significant QTL regions are presented in Additional file 9: Table S8. Result from the haplotype-based GWAS was generally consistent with those from SNP-based GWAS, with only one significant QTL region (169.7–170.7 Mb) identified, on GGA1 (Fig. 2b).

**Fig. 3:**
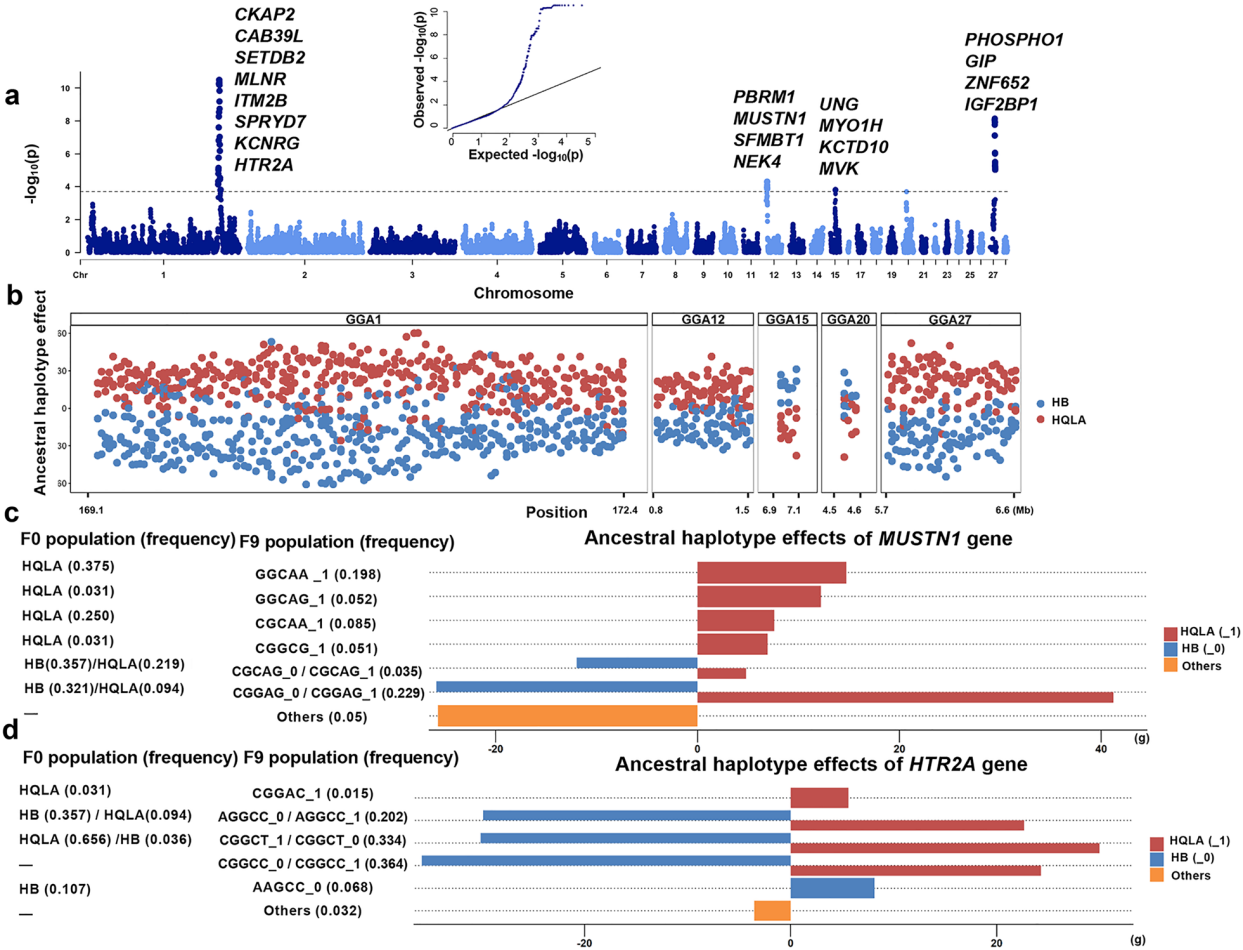
Results from the ancestral-haplotype-based GWAS and ancestral haplotype analysis. **a** Results from ancestral-haplotype-based GWAS, candidate genes overlapping with the peaks are presented (The dashed black horizontal line shows the FDR < 5% cutoff). **b** Estimates of effects of haplotypes of different ancestral origins in major QTLs. Red points indicate haplotypes from the HQLA population; blue points indicate haplotypes from the HB population. **c** Ancestral haplotype analysis for blocks that cover the *MUSTN1* gene. Six haplotypes were retrieved from the HB population (nhap = 2) and the HQLA population (nhap = 6) with haplotype frequencies > 0.05. Red bars (_1) indicates haplotypes exclusive to the HQLA population, blue bars (_0) indicates haplotypes exclusive to the HB population, orange bars indicate haplotypes absent in both populations.** d** Ancestral haplotype analysis for blocks covering the *HTR2A* gene. Four haplotypes were retrieved from the HB population (nhap = 3) and the HQLA population (nhap = 2) with haplotype frequencies > 0.05

We subsequently delved into the significant genomic region (GGA1: 170,559,701 bp) identified in both the SNP-based GWAS and the haplotype-based GWAS. This block contained 10 distinct haplotype alleles, with frequencies ranging from 0.001 to 0.436. Using a separate mixed model, the effect size of each haplotype allele in this block on BW8 phenotype was estimated to range from – 40.4 to 42.8 g. Most haplotype alleles with negative effects were uniquely transmitted from the HB population (Fig. 2c). The haplotype TTAGG that showed the highest positive effect was present in both the HB and the HQLA population but its frequency was much higher in the HQLA population (0.969) than in the HB population (0.036). Given the genetic homogeneity of the F_9_ population, alongside the differentiated haplotype structures of the HB and HQLA populations, coupled with their distinct bodyweight phenotypes, it is plausible that the haplotype effects observed in the F_9_ generation were associated, to some extent, with their ancestral origins. We, therefore, conducted an ancestral-haplotype-based GWAS by grouping haplotypes according to their ancestral origins. RFMix was used to determine the ancestral origin of each haplotype in the F_9_ population. The estimated ancestries of haplotypes from the F_9_ population were about 1:1 from the HB and HQLA populations, concordant with the random mating strategy employed in AIL construction (see Additional file 10: Fig. S2a). To empirically assess the accuracy of the ancestry estimation, we checked the estimated ancestries of haplotypes that were unique in the HQLA or the HB population and found an approximate accuracy of 0.951 (see Additional file 10: Fig. S2b). Compared with results from SNP-based GWAS, the ancestral-haplotype-based GWAS identified the same signals on GGA1: H6770–H6906 (169,110,052–172,421,963 bp) and GGA27: H121-H154 (5,717,631- 6,575,040 bp). Additionally, ancestral-haplotype-based GWAS revealed signals for successive blocks on GGA12: H21–H33 (1,121,219–1,422,421 bp); GGA15: H261–H263 (6,947,970–7,036,413 bp); and GGA20: H184-H187 (4,510,669–4,629,756 bp) (see Fig. 3a and Additional file 11: Table S9). Statistics and corresponding genes of these newly identified significant blocks are listed in Additional file 12: Table S10. For most blocks, haplotypes derived from the HQLA population exhibited positive effects on BW8. However, for a few blocks on GGA15 and GGA20, the direction was opposite, with haplotypes derived from the HB population displaying positive effects (Fig. 3b). Signals on GGA27 were notably prominent compared to the corresponding signals in the SNP-based GWAS, indicating that ancestral-haplotype-based GWAS successfully assesses the collective effects of haplotypes within these blocks, by grouping them according to their ancestral origins.

One gene, *MUSTN1,* located on GGA12 (GGA12:1,237,478–1,240,970 bp) was previously reported to play an important role in skeletal-muscle growth in chicken [42]. In the block covering the *MUSTN1* gene, the estimation of effect sizes for haplotype alleles showed that all five haplotypes that originated from the HQLA population had positive effects on body weight. Among them, four haplotype alleles, namely, CGCAA, CGGCG, GGCAA, and GGCAG, were unique to the HQLA population. Haplotype alleles CGCAG and CGGAG originated from both populations. For these, the HB-origin haplotypes exhibited negative effects, while the HQLA-origin haplotypes showed positive effects (Fig. 3c).

Another interesting candidate is gene *HTR2A*, located on GGA1 (GGA1: 169,670,496–169,697,156 bp), which has been previously shown associated with growth and development in chicken [43]. Again, haplotype alleles with identical sequences but different ancestral origins exhibited clearly opposite effects, affirming the complex genetic background of the growth trait (Fig. 3d). One haplotype allele that was unique to the HB population, AAGCC, exhibited positive effects on body weight, indicating that some beneficial haplotype alleles were “hidden” in the low-body-weight HB population (Fig. 3d). Detailed ancestral haplotype analysis with effect size estimation and their corresponding genes are listed in Additional file 13: Table S11.

### Ancestral-based dominance in F_9_ population

Although non-additive effects are generally considered to be not stably inherited in subsequent generations, genetic interactions from different ancestral backgrounds may still be active even after many generations during breed formation. The AIL population provides a unique opportunity to test ancestral-based dominance, as it was constructed by two chicken populations with distinct genetic backgrounds.

**Fig. 4:**
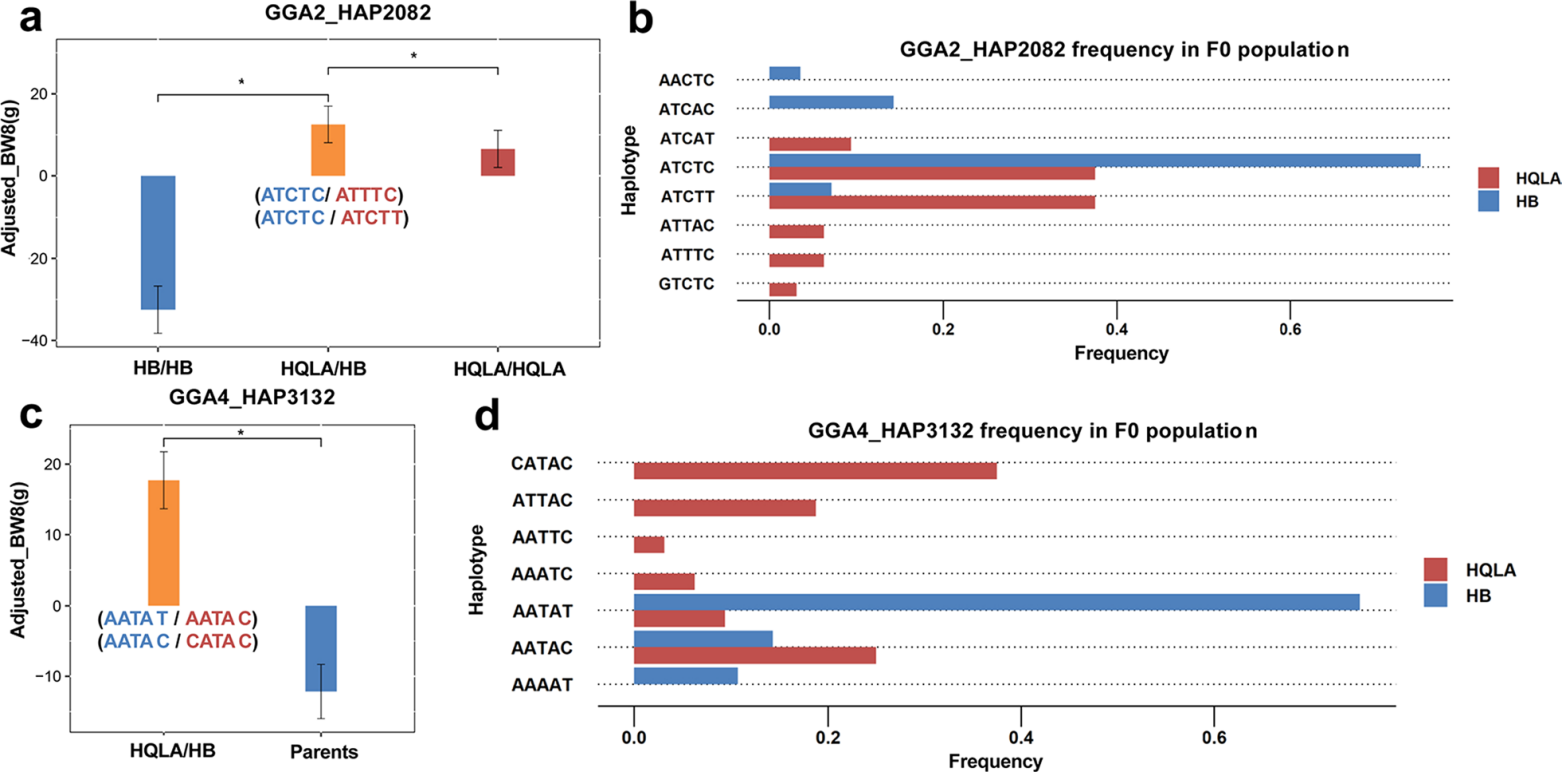
Analysis of dominance in the F_9_ population. **a** Example of high-parent dominance on haplotype GGA2_HAP2082. The red and blue sequences in parentheses indicate the haplotypes from HQLA and HB populations, respectively. **b** Haplotype allele frequency in two ancestral populations for block GGA2_HAP2082. **c** Example of mid-parent dominance on GGA4_HAP3132. The red and blue sequences in parentheses indicate the haplotypes from HQLA and HB populations, respectively. **d** Haplotype allele frequency in the two ancestral populations for block GGA4_HAP3132

As the body weight of the founders from HB or HQLA was not recorded, we explored high-parent dominance in the F_9_ population for loci for which the ancestral heterozygote exhibited significantly higher body weight compared to any of the ancestral homozygotes. Eight blocks on GGA2 passed the Kruskal–Wallis tests (see Additional file 14: Table S12). The average Jensen-Shannon divergence of the F_0_ population for these eight blocks was 0.167 ± 0.099, comparable to the genome average. It is worth noting that, based to our definition, the ancestral heterozygote can include two identical haplotype alleles but with distinct origins. In addition, for none of the eight blocks were each haplotypes completely unique to one population. This contrasts with inbreeding in plants, but consistent with the heterogeneous haplotype structure [44] that was revealed in the preceding section. Four known protein-coding genes were annotated for the eight blocks. As an example, Fig. 4a, b show the exact superior ancestral heterozygote, and the haplotype allele frequencies in the F_0_ population for block HAP2082 on GGA2. The candidate gene *KBTBD2* (Kelch repeat and BTB domain containing 2) in haplotype HAP2082 belongs to the Kelch protein family, which has an effect on skeletal-muscle development [45].

We used a relaxed criterion to identify loci with mid-parent dominance and this resulted in identification of 624 blocks with mid-parent effects across 12 chromosomes (see Additional file 15: Table S13). The average Jensen-Shannon divergence of the F0 population for the 624 blocks was 0.217 ± 0.150. Since the average Jensen-Shannon divergence for loci that exhibited high-parent and mid-parent dominance was not higher than the genome average, dominance does not necessarily covary with genetic distance. We used one block (HAP3132) on GGA4 as an example (Fig. 4c, d), which includes the *KCNIP4* gene (*potassium voltage-gated channel interacting protein 4*). *KCNIP4* has extensive physiological functions, including neurotransmitter release, smooth muscle contraction, heart-rate adjustment, and insulin secretion, and it was also associated with growth traits in a different chicken population [46].

### Prioritization of candidate genes associated with body weight

To obtain a reduced set of best candidate genes, we prioritized 220 candidate genes reported by GWAS and dominance analyses using the ToppGene web service (https://toppgene.cchmc.org), using known genes associated with human body weight as the training set from the GWAS Catalog. We successfully prioritized 185 genes (see Additional file 16: Table S15), including 12 genes identified by ancestral-haplotype-based GWAS, 4 genes identified by high-parent dominance analysis, and 169 genes identified by mid-parent dominance analysis. Table 2 listed the top 10 genes based on prioritized p values.

**Table 2:** Top prioritized positional candidate genes for body weight

Gene	Chromosome	Position (bp)	Rank	P value
*EYA1*	GGA2	116,925,618–117,074,063	1	3.286E−04
*PDE1C*	GGA2	48,126,705–48,416,766	2	1.015E−03
*MYC*	GGA2	139,734,098–139,738,744	3	2.704E−03
*NCOA2*	GGA2	116,504,849–116,596,399	4	8.694E−03
*EDNRA*	GGA4	31,904,342–31,933,857	5	9.259E−03
*CREBBP*	GGA14	12,891,875–12,969,501	6	9.550E−03
*DACH2*	GGA4	8,277,517–8,541,470	7	1.283E−02
*ANK2*	GGA4	56,725,180–57,009,349	8	1.544E−02
*MAB21L2*	GGA4	33,007,990–33,009,211	9	1.737E−02
*KIF26B*	GGA3	34,279,395–34,717,416	10	2.256E−02

Among the best candidate genes, the *EYA1* gene (GGA2: 116,925,618–117,074,063) encodes a protein that plays roles in the development of eyes and ears. Mutations in this gene were reported to cause stunted growth and slowed development in frog [47]. *PDE1C* (GGA2: 48,126,705–48,416,766 bp) encodes an enzyme that regulates the proliferation and migration of vascular smooth muscle cells, and neointimal hyperplasia. Previous studies have shown its relationship to Type 2 diabetes in humans [48]. *MYC* (GGA2: 139,734,098–139,738,744 bp) is a transcription factor that has been described to enhance the expression of growth-promoting genes in human [49].

## Discussion

AIL constitute a valuable resource for mapping quantitative traits with high resolution as a resulted of accumulated genome recombination. They are commonly used in animal genetics research [50, 51]. The F_9_ AIL used in this study is a segregating population created by the random intercrossing populations over nine-generations. The initial parental population originated from the HB and HQLA populations, which have distinct phenotypes and genetic backgrounds. The HB chicken is a local Chinese breed that has not been subject to strong artificial selection. The HQLA population is a closed broiler population that has been under strong artificial selection for body weight for more than 10 generations.

Populations with extreme phenotypes often exhibit significant genetic differences due to prolonged geographic isolation or different selection strategies. The genetic architecture of chicken body weight is complex, involving many genes with small effects that collectively contribute to the phenotype [52, 53]. However, due to the highly heterogeneous genetic architecture, selection struggles to drive a particular allele to fixation, which poses challenges for effective association mapping.

In this study, we developed an analytic strategy to assess the collective genetic contributions of haplotypes with different ancestral origins to phenotypic variation, motivated by the observation that haplotypes of different ancestral origins in our AIL population carried distinct effects in both our recent [6] and current studies. The ancestral-haplotype-based GWAS was first used for preliminary screening for association signals. Subsequently, we carried out haplotype analysis for each significant locus to analyze the detailed effects of haplotype alleles. Instead of clustering haplotypes by sequence, we proposed to cluster haplotypes on the basis of ancestral origin. By leveraging ancestral information, our method efficiently revealed that haplotypes originating from the HB and HQLA population generally had positive or negative effects respectively. Our ancestral-haplotype-based GWAS avoids the problem of reduced power because of excessive degrees of freedom in the haplotype analysis. The application of ancestral-haplotype-based GWAS can extend to other populations, provided the ancestral population is known, and the ancestral origin of the mixed individual can be inferred. In our study, RFmix was used to trace the origin of haplotypes from the two ancestral populations. It is feasible to apply our method to multiple ancestral populations by using RFMix or other local ancestry inference tools. Our ancestral-haplotype-based GWAS reported signals on GGA1 and GGA27, which were also detected by a standard SNP-based GWAS approach. However, it also reported several new signals on GGA12, GGA15, and GGA20, which were further annotated as biologically relevant. Follow-up haplotype analysis identified specific haplotype alleles with considerable effect sizes in the F_9_ population that can be used as starting points to improve breeding efficiency. The strength of ancestral-haplotype-based GWAS lies in the fact that haplotypes originating from the HB and the HQLA population frequently have different directionality of their effects. However, it should be noted that if trait-increasing and trait-decreasing haplotypes are comparable in number within a population, it would result in a substantial loss of power.

By incorporating ancestral information, our GWAS strategy naturally detected ancestry-based dominance. The non-additive analysis we employed here is basically of genetic interactions of haplotype alleles from different ancestral backgrounds. We identified many candidate loci with statistical support. While some candidates have been functionally validated in previous studies [45, 46], further research is required to understand the genes or haplotypes responsible for dominance on chicken growth and their underlying mechanisms.

Body weight at eight weeks of age is one of most important economic traits in the chicken industry. Despite many efforts to fine-map traits in chicken using AIL populations [54–56], loci with small effects are left undetected. By employing ancestral-haplotype-based GWAS, we reported several new candidate genes for the existing population. Ubiquitin protein ligase E3B (*UBE3B*), located in GGA15: 7,041,591–7,058,666 bp, controls water holding capacity in pigs [57], which affects loss of tissue fluid, and in turn weight loss. The beneficial haplotype alleles originated from HB and had a frequency of about 92.9% in the HB F_0_ population. This suggests that, for complex traits, candidate genes might be related to overlooked sub-phenotypes, such as water-holding capacity and its impact on weight. Those results underscore the importance of considering sub-phenotypes in genetic studies and the value of ancestral-haplotype-based GWAS in uncovering novel genetic markers for complex traits.

## Conclusions

In this study, we introduced analytical strategies that integrate haplotype analysis with ancestral origins in AIL populations. Through this approach, we identified novel associations for chicken body weight at eight weeks of age on GGA12, GGA15, and GGA20 in the F_9_ AIL population. By incorporating ancestral information, we applied concepts of ancestral homozygotes and ancestral heterozygotes at haplotypes. We identified genetic loci that exhibited high-parent and mid-parent dominance for chicken body weight. Finally, we prioritized candidate genes, highlighting *EYA1*, *PDE1C* and *MYC* as the best candidates for further validation. Our results contribute to a better utilization of the AIL population for genetic mapping.

## Supplementary Information

Additional file 1: Table S1. Comparison of phasing results between SHAPEIT and Beagle software.

Additional file 2: Table S2. A set of 324 genes associated with human body weight from GWAS Catalog.

Additional file 3: Figure S1. Comparison of polygenic structure between different populations (GGA15-GGA28). a) Counting unique haplotypes in different populations. b) Distribution of H12, H123, and H1234 statistics in different populations. c) Distribution of Jaccard distance of F0 and F9 populations "*" indicates the mean value.

Additional file 4: Table S3. The genetic map of chicken.

Additional file 5: Table S4. Summary statistics for the genetic map and number of informative markers in the F9 population.

Additional file 6: Table S5. The result of standard SNP-based GWAS for body weight at eight weeks.

Additional file 7: Table S6. The significant loci of SNP-based GWAS.

Additional file 8: Table S7. The results of haplotype-based GWAS.

Additional file 9: Table S8. The significant loci of haplotype-based GWAS.

Additional file 10: Figure S2. The results of RFMix. a) Distribution of probability of haplotypes derived from the HQLA population. If the value of probability is less than 0.5, the haplotypes was considered to be of HB origin; otherwise, the haplotypes was considered to be of HQLA origin. b) Distribution of inference accuracy of RFMix.

Additional file 11: Table S9. The results of ancestral-haplotype-based GWAS.

Additional file 12: Table S10. New signals in GGA12, GGA15, GGA20.

Additional file 13: Table S11. Significant ancestral haplotype analysis with effect size estimation and their corresponding genes.

Additional file 14: Table S12. 11 Blocks of high-parent dominance.

Additional file15: Table S13. Blocks of mid-parent dominance across 12 chromosomes.

Additional file 16: Table S14. Prioritized genes associated with body weight.

Additional file 17: Script information used in this study.

## Data Availability

The raw sequence reads are from the SRA database (SRA accession: SRP079718). Scripts for analyses used in this study are available in Additional file 17.
