# Supplementary figures and images for: Haplotype analysis incorporating ancestral origins identified novel genetic loci associated with chicken body weight using an advanced intercross line
Source: Genet Sel Evol. 2024 Dec 20;56:78. doi: 10.1186/s12711-024-00946-y (PMC11660596; doi:10.1186/s12711-024-00946-y)

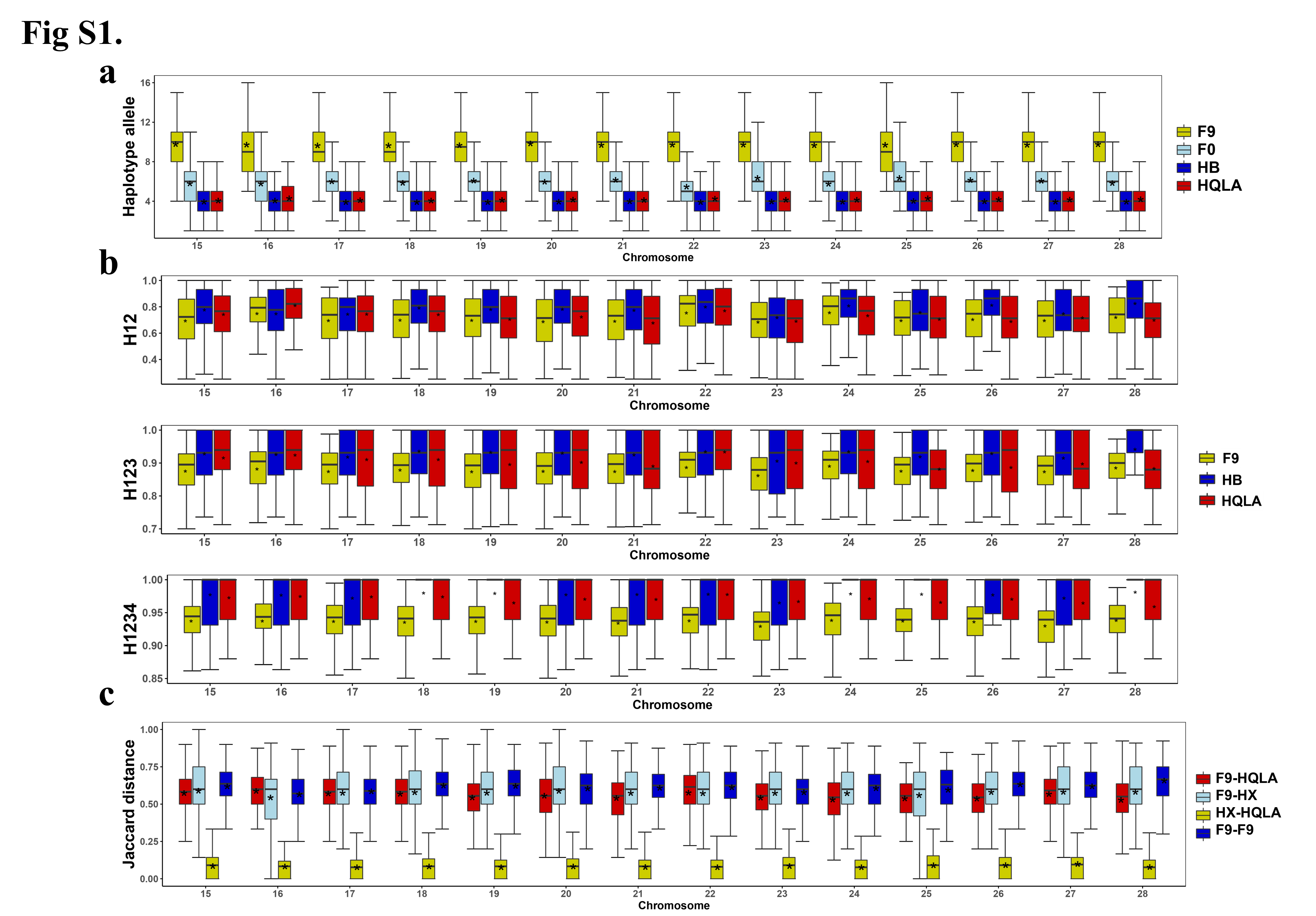

Supplement: Supplementary file 3 — Additional file 3: Figure S1. Comparison of polygenic structure between different populations (GGA15-GGA28). a) Counting unique haplotypes in different populations. b) Distribution of H12, H123, and H1234 statistics in different populations. c) Distribution of Jaccard distance of F0 and F9 populations "*" indicates the mean value. [file 12711_2024_946_MOESM3_ESM.docx]

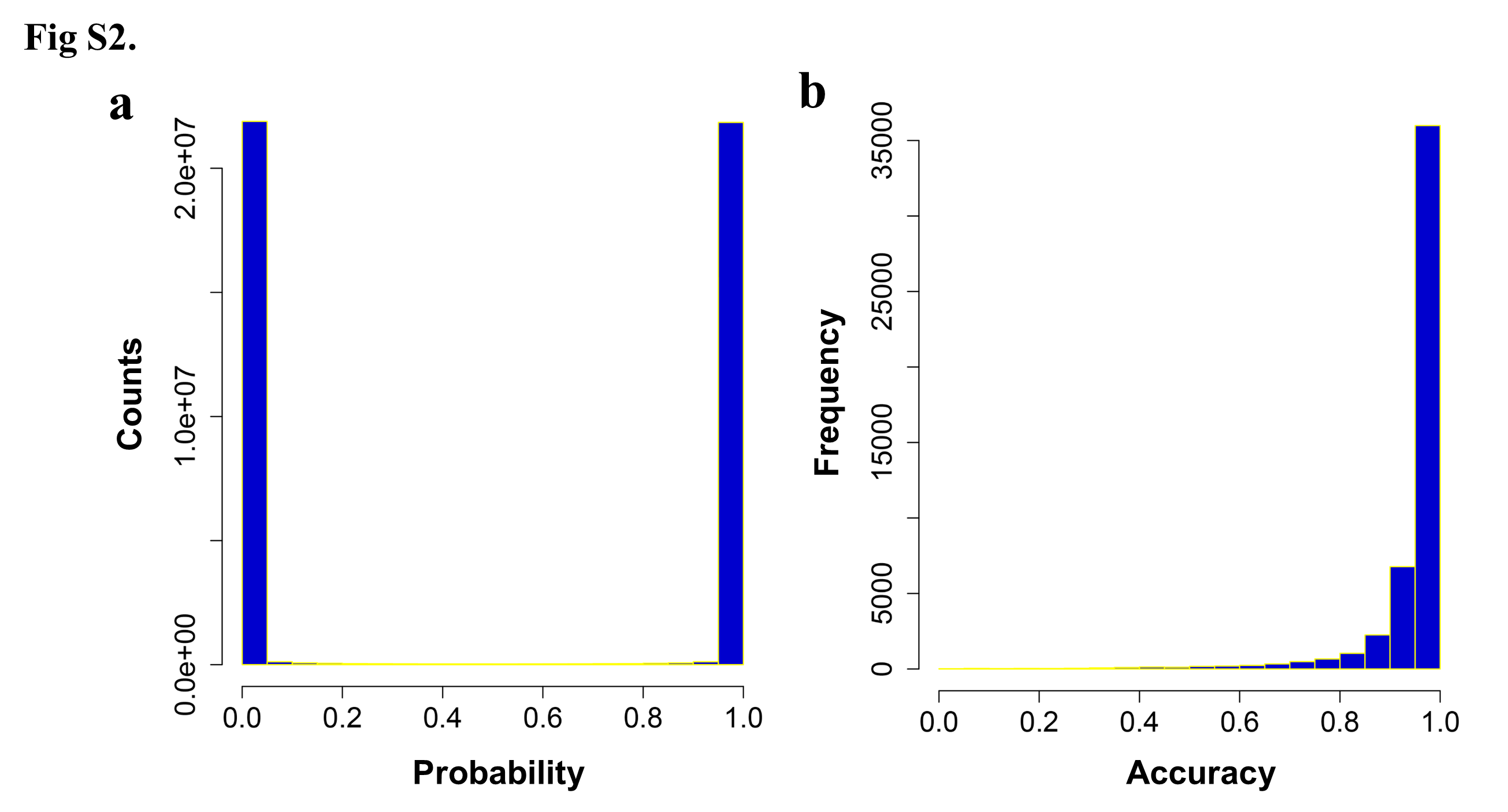

Supplement: Supplementary file 10 — Additional file 10: Figure S2. The results of RFMix. a) Distribution of probability of haplotypes derived from the HQLA population. If the value of probability is less than 0.5, the haplotypes was considered to be of HB origin; otherwise, the haplotypes was considered to be of HQLA origin. b) Distribution of inference accuracy of RFMix. [file 12711_2024_946_MOESM10_ESM.docx]
